# Ti-Doped, Mn-Based Polyanionic Compounds of Na_4_Fe_1.2_Mn_1.8_(PO_4_)_2_P_2_O_7_ for Sodium-Ion Battery Cathode

**DOI:** 10.3390/nano15080581

**Published:** 2025-04-11

**Authors:** Hualin Li, Gang Pang, Weilong Zhang, Qingan Zhang, Linrui Hou, Changzhou Yuan

**Affiliations:** 1 School of Materials Science and Engineering, Key Laboratory of Efficient Conversion and Solid-State Storage of Hydrogen & Electricity of Anhui Province, Anhui University of Technology, Ma’anshan 243002, China; lihualin@stu.ahut.edu.cn (H.L.); tanjie@ahut.edu.cn (W.Z.); 2 School of Materials Science & Engineering, University of Jinan, Jinan 250022, China; mse_houlr@ujn.edu.cn

**Keywords:** sodium-ion batteries, Na_4_Fe_1.2_Mn_1.8_(PO_4_)_2_P_2_O_7_, Mn-doped, Ti-doped, electrochemical performances

## Abstract

Na_4_Fe_3_(PO_4_)_2_P_2_O_7_ (NFPP) is recognized as a prospective electrode for sodium-ion batteries (SIBs) because of its structure stability, economic viability and environmental friendliness. Nevertheless, its commercialization is constrained by low operating voltage and limited theoretical capacity, which result in a power density significantly inferior to that of LiFePO_4_. To address these limitations, in this work, we first designed and synthesized a series of Mn-doped NFPP to enhance its operating voltage, inspired by the successful design of LiFe_1-*x*_Mn*_x_*PO_4_ cathodes. This approach was implemented to enhance the operating voltage of the material. Subsequently, the optimized Na_4_Fe_1.2_Mn_1.8_(PO_4_)_2_P_2_O_7_ (1.8Mn-NFMPP) sample was selected for further Ti-doped modification to enhance its cycle durability and rate performance. The final Mn/Ti co-doped Na_4_Fe_1.2_Mn_1.7_Ti_0.1_(PO_4_)_2_P_2_O_7_ (0.1Ti-NFMTPP) material exhibited a high operating voltage of ~3.6 V (vs. Na^+^/Na) in a half cell, with an outstanding reversible capacity of 122.9 mAh g^−1^ at 0.1 C and remained at 90.6% capacity retention after 100 cycles at 0.5 C. When assembled into a coin-type full cell employing a commercial hard carbon anode, the optimized cathode material exhibited an initial capacity of 101.7 mAh g^−1^, retaining 86.9% capacity retention over 50 cycles at 0.1 C. These results illustrated that optimal Mn/Ti co-doping is an effective methodology to boost the electrochemical behavior of NFPP materials, achieving mitigation of the Jahn–Teller effect on the Mn^3+^ and Mn dissolution problem, thereby significantly improving structural stability and cycling performance.

## 1. Introduction

With the increasing attention to the environmental problems attributed to fossil energy, the rapid development of sustainable energy requires advanced energy storage systems with economic feasibility, energy efficiency and reliability [1,2]. Lithium-ion batteries (LIBs) are extensively employed in electronic devices owing to their high energy density. However, limited lithium resources, which are unevenly dispersed in the Earth’s crust, and the high cost of lithium may result in shortage with the further application of LIBs for electric vehicles (EV) [3,4]. Similar to the energy storage mechanism of LIBs, SIBs have drawn considerable research attention owing to the abundance and wide distribution of sodium sources [5,6,7]. Additionally, sodium has a higher standard electrode potential compared to lithium, which indicates that aluminum can serve as the electric current collector for the anodes in SIBs [8,9,10]. Therefore, SIBs are viewed as a possible cheaper, safer and more sustainable alternative to LIB technology for large-scale applications.

Recently, tremendous efforts have been made in the developments of SIB techniques, which have a number of drawbacks to be used as an alternative to LIBs systems, such as the low reversible sodiation/desodiation kinetics and limited capacity [11,12,13]. For this reason, a series of electrode materials designed for SIBs have been developed to meet the special requirements of the host material’s electrochemical reaction. For the positive electrode materials, layered metal oxides [14,15] (including P2 type and O3 type), Prussian blue and its analogs [16,17] (Na_1+*x*_M_1_[M_2_(CN)_6_]∙*z*H_2_O, *x* = 1~2 and M_1_ = Ni, Cu, Zn, etc., which is the transition-metal ion linked with N; and M_2_ is the metal ion coordinated with C) and polyanionic compounds [18,19] (phosphate, sulfate, fluorophosphates, pyrophosphates, mixed polyanions, etc.) are widely investigated. Among the above-mentioned three types of cathode materials, mixed polyanionic compounds have emerged as prime candidates for SIBs in advanced energy storage applications, due to their exceptional structural stability and ultra-long cycling performance [20,21]. Over the past decade, extensive research about the iron-based mixed polyanionic cathodes, NFPP, have demonstrated exceptional electrochemical sodium-storage performance, which are being mass-produced due to low cost, non-toxic properties and a synthesis process similar to LiFePO_4_ [22,23]. However, the theoretical capacity and operating voltage of NFPP are only 129 mAh g^−1^ and 3.0 V (vs. Na^+^/Na), respectively, resulting in approximately 110 Wh Kg^−1^ of energy density that is far inferior to that of LiFePO_4_ [24,25,26].

Learning that LiFe_1-*x*_Mn*_x_*PO_4_, by doping Mn into LiFePO_4_, increases the voltage platform from 3.2 V to 4.1 V (vs. Li^+^/Li), consequently leading to higher energy density, some researchers hope to increase the voltage plateau of NFPP by replacing the partial Fe with Mn or other elements to improve its energy density [27,28,29,30,31,32,33]. Ma et al. reported on Mn-doped Na_4_Fe_3-*x*_Mn*_x_*(PO_4_)_2_P_2_O_7_/rGO (*x* = 0, 0.15, 0.30 and 0.45), and found Mn^2+^ substitution effectively reduces the Na^+^ diffusion activation energies and decreases the intrinsic band gap of the crystal lattice by density functional theory calculation. Meanwhile, the most favorable Na_4_Fe_2.7_Mn_0.3_(PO_4_)_2_P_2_O_7_/rGO achieved a capacity of 131.5 mAh g^−1^ at 0.1 C with notable capacity retention and exceptional low-temperature performance at −20 °C [34]. Li et al. prepared the carbon-coat Mn^2+^ doping Na_4_Fe_2.9_Mn_0.1_(PO_4_)_2_P_2_O_7_/C via mechanochemical synthesis with its capacity of 119.6 mAh g^−1^ at 0.1 C and 84.8% retention after 3000 cycles under high-rate 10 C [35]. Pu^’^s team systematically reported on the Mn-doped Na_4_Fe_3-*x*_Mn*_x_*(PO_4_)_2_P_2_O_7_ (*x* = 0.3, 0.6, 0.9, 1.2, 1.5, 1.8 and 2.1) cathodes and revealed that moderate Mn substitution (0.6 ≤ *x* ≤ 1.2) can effectively weaken the Fe-O bonding interaction, leading to the full utilization of the Mn^3+^/Mn^2+^ redox couple. Particularly, the optimized Na_4_Fe_1.8_Mn_1.2_(PO_4_)_2_P_2_O_7_ cathode exhibits a high voltage of ~3.3 V (vs. Na^+^/Na), exceeding that of the pristine NFPP, along with a capacity of 109.2 mAh g^−1^, favorable rate performance and remarkable cycle durability [36]. The above researchers have primarily utilized a single Mn-doping approach to optimize the electrochemical properties of NFPP, while Wang developed a high-entropy modification of multiple elements strategy to further improve its properties. The optimized sample of Na_3.9_Fe_2.6_V_0.1_Mn_0.1_Cu_0.1_Mg_0.1_(PO_4_)_2_P_2_O_7_ demonstrates impressively superior capacity of 122.3 mAh g^−1^ at 0.1 C with an insertion/extraction of 2.8 Na^+^, sustaining excellent performance over 14,000 cycles at 50 C [37]. Therefore, drawing inspiration from the design of the LiFe_1-*x*_Mn*_x_*PO_4_ cathode, the Mn-doped NFPP composition has been established as one of the effective methods for improving its energy density.

In this work, we first designed and synthesized a series of Mn-doped Na_4_Fe_3-*x*_Mn*_x_*(PO_4_)_2_P_2_O_7_/C (denoted as *x*Mn-NFMPP, *x* = 1.5, 1.8, 2.1, 2.4 and 2.7), based on the Mn/Fe molar ratio in current commercial LiFe_1-*x*_Mn*_x_*PO_4_ cathodes which typically exceeds 6:4, increasing the Mn content in LiFe_1-*x*_Mn*_x_*PO_4_ and expecting to enhance their operating voltage. Unfortunately, the Mn-rich phosphate cathode materials still encounter intrinsically poor electronic conductivity and inevitable structural deterioration and non-reversible phase transition arising from the Jahn–Teller distortion throughout charge–discharge processes, thus deteriorating the specific capacity, cycling and rate performance [38]. Previously reported Mn-based transition metal oxides (Na_0.44_[Mn_1-*x*_Ti*_x_*]O_2_ [39], Na_0.61_[Mn_0.61-*x*_Fe*_x_*Ti_0.39_]O_2_ [40], P2-type Na_0.67_Fe_0.5-*x*/2_Mn_0.5-*x*/2_Ti*_x_*O_2_ [41] and O3-type NaLi_1/3_Ti_1/6_Mn_1/2_O_2_ [42]) and polyanionic cathodes (Li(Mn_0.85_Fe_0.15_)_0.92_Ti_0.08_PO_4_ [43], Na_3_V_1.9_Ti_0.1_(PO_4_)_3_ [44], Na_3_TiMn(PO_4_)_3_ [45] and Na_3.4_Mn_1.2_Ti_0.8_(PO_4_)_3_ [46]) revealed Ti doping can alleviate the Jahn–Teller effect of the Mn^3+^ and Mn dissolution problem. Consequently, the Na_4_Fe_1.2_Mn_1.8_(PO_4_)_2_P_2_O_7_/C in the aforementioned report synthesized various materials and optimized these, and the optimized sample was selected for further Ti-doped modification to suppress the Mn^3+^-induced octahedral distortions and Mn dissolution problem, thereby offering potential improvements in the material’s electrochemical properties. Based on the above analysis, we further synthesized a series of Ti-doped Na_4_Fe_1.2_Mn_1.8-*x*_Ti*_x_*(PO_4_)_2_P_2_O_7_/C (denoted as *x*Ti-NFMTPP, *x* = 0.1, 0.15, 0.2 and 0.3) materials using the same synthetic method. The results show that Ti-doped Na_4_Fe_1.2_Mn_1.8-*x*_Ti*_x_*(PO_4_)_2_P_2_O_7_/C exhibits a superior specific capacity together with remarkable cycling stability even at high current densities. Typically, the Na_4_Fe_1.2_Mn_1.7_Ti_0.1_(PO_4_)_2_P_2_O_7_/C cathode exhibits an enhanced voltage of ~3.6 V (vs. Na*^+^*/Na) in a half cell, much higher than that of NFPP. The cathode demonstrated the highest capacity of 122.9 mAh g^−^¹ at 0.1 C and maintained 90.6% of its initial capacity over 100 cycles at 0.5 C. The assembled hard carbon||Na_4_Fe_1.2_Mn_1.7_Ti_0.1_(PO_4_)_2_P_2_O_7_/C coin-type full cell achieved an initial capacity of 101.7 mAh g^−1^ when tested at 0.1 C, maintaining 86.9% capacity after 50 cycles.

## 2. Experimental Section

### 2.1. Materials Synthesis

Analytical-grade chemicals employed in this study were entirely obtained from Sinopharm Chemical Reagents Corporation (Shanghai, China). The designed *x*Mn-NFMPP (*x* = 1.5, 1.8, 2.1, 2.4 and 2.7) materials were fabricated via a spray-dried technique with subsequent high-temperature heat treatment. Firstly, stoichiometric amounts of Mn(CH_3_COO)_2_·4H_2_O, Fe(NO_3_)_3_⋅9H_2_O and C_6_H_8_O_7_⋅H_2_O were separately dissolved in 500 mL of 0.5 M nitric acid solution. Subsequently, a stoichiometric amount of NH_4_H_2_PO_4_ and Na_4_P_2_O_7_ added to 100 mL deionized water were slowly introduced via dropwise infusion into the target solution with continuous agitation for 2 h. The excess stoichiometry of 3 mol% Na_4_P_2_O_7_ was considered to compensate for Na and P evaporation during high temperatures. Then, the homogenized solution underwent spray-drying at 200 °C to yield powder precursors. Finally, the precursors were annealed at 300 °C for 6 h and afterwards at 650 °C for 10 h with 2 °C min^−1^ in a N_2_ atmosphere. The *x*Ti-NFMTPP (*x* = 0.1, 0.15, 0.2 and 0.3) samples were fabricated with the same method mentioned above, except for the addition of C_16_H_36_O_4_Ti as a Ti dopant.

### 2.2. Material Characterization

The X-ray diffraction (XRD) patterns were collected by the BRUKER D8 diffractometer (Bruker, Billerica, MA, USA) (Cu Kα radiation) along with Rietveld refinement using the RIETAN-FP program [47]. The microstructural properties were examined by scanning electron microscopy (SEM) (Nova Nano 430, FEI, Hillsboro, OR, USA) and transmission electron microscopy (TEM) coupled with energy dispersive X-ray spectroscopy (EDS) (JEM-210, JEOL, Tokyo, Japan). Carbon content was measured with a thermal gravimetric (TG) analyzer (NETZSCH STA 409 PC, NETZSCH, Selb, Germany) with 5 °C min^−1^ from 50~800 °C under air. The Raman spectra were acquired by a Raman spectrometer (LabRAM Soleil, HORIBA, Lyon, France) to analyze the carbon coating within 1000~1700 cm^−1^. The X-ray photoelectron spectroscopy (XPS) characterization was conducted using a Perkin-Elmer PHI 550 spectrometer (PerkinElmer, Waltham, MA, USA) with Al Kα. The Fourier-transform infrared spectroscopy (FT-IR) was conducted with an FT-IR Spectrometer (FT-IR-4100, JASCO, Easton, MD, USA) using the transmission technique with KBr pellets.

### 2.3. Electrochemical Characterization

The electrochemical properties were tested with 2032 coin-type half cells. Cathode mixtures were formulated by blending the prepared materials (85 wt.%), polyvinylidenefluoride (PVDF 5 wt.%) and acetylene black (10 wt.%) in N-methyl-2-pyrrolidone (NMP). These mixtures were spread evenly onto carbon-coated Al current collectors with a 12 mm diameter and vacuum-dried for 24 h at 110 °C. The active material loading on the cathode was about 2.0 mg. The cells were constructed with glass fiber membranes and metallic sodium electrodes in an Ar atmosphere. The electrolytic solution contained 1.0 M NaClO_4_ in a dimethyl carbonate (DMC)/ethylene carbonate (EC) (1:1 by volume) mixture with 5 vol.% of fluoroethylene carbonate (FEC). The electrochemical experiments were carried out across various current densities within 1.5~4.4 V (vs. Na^+^/Na) employing CT2001A instrumentation. Cyclic voltammetry (CV) profiles were collected with a CHI 660E analyzer (CH Instruments Ins., Austin, TX, USA).

## 3. Result and Discussion

**Figure 1:**
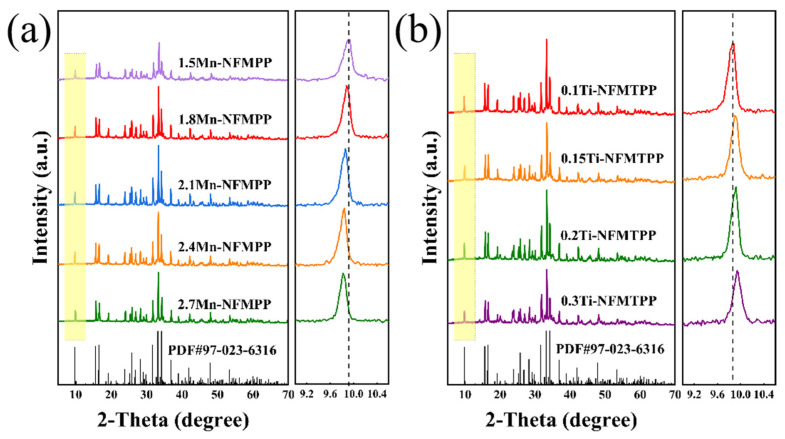
XRD patterns and local magnification of (**a**) *x*Mn-NFMPP (*x* = 1.5, 1.8, 2.1, 2.4 and 2.7) and (**b**) *x*Ti-NFMTPP (*x* = 0.1, 0.15, 0.2 and 0.3), respectively.

Figure 1a,b illustrates the XRD patterns of *x*Mn-NFMPP (*x* = 1.5, 1.8, 2.1, 2.4 and 2.7) and *x*Ti-NFMTPP (*x* = 0.1, 0.15, 0.2 and 0.3) samples. The samples had very sharp diffraction peaks of a well-crystallized NFPP phase with the orthorhombic *Pn*2_1_*a* (33) space group, which can be exclusively indexed to the reference pattern of PDF No. 97-023-6316 for the NFPP phase. No other impurity phases could be detected, such as NaFePO_4_ and/or Na_2_FeP_2_O_7_, revealing the purity of these samples. In Figure 1a, the XRD patterns illustrated that the varying Mn-doped content exerted negligible influence on the phase purity of the mixed polyanion compounds, which could be primarily attributed to the isostructural framework of the Na_4_M_3_(PO_4_)_2_P_2_O_7_ (M = Ni, Co, Mn, Fe) compounds [48], and allowed for the practical in situ substitution of Mn for Fe. This inherent crystallographic compatibility enables effective solid–solution formation through isovalent cation exchange, particularly facilitating the substitution of Fe^2^^+^ ions by Mn^2^^+^ dopants, which was consistent with those observed in other Mn-doped NFPP [49]. With the elevated content in Mn substitution, the diffraction peaks gradually migrated toward lower angles, indicative of the increase in the interplanar spacing, mainly attributed to the replacement of Fe^2+^ (0.74 Å) by Mn^2+^ (0.80 Å) with a larger ionic radius in the host structure. The Rietveld refinement was further proven to quantify the crystal lattice parameters of the XRD data, as displayed in Figures S1–S5 (Supplementary Information), and the resulting calculated parameters are tabulated within Table S1. Evidently, the refinement date revealed that the lattice parameters (a, b, c) and unit-cell volume (V) exhibited a gradually increase when the Mn substitution ratio increased. With the elevated content in Mn substitution, the unit-cell volume of the *x*Mn-NFMPP materials progressively expanded from 1273.03(8) Å^3^ (1.5Mn-NFMPP) to 1280.44(1) Å^3^ (2.1Mn-NFMPP), and then reaching 1288.99(8) Å^3^ (2.7Mn-NFMPP). The systematic evolution of the crystalline structure parameters along with the Mn substitution ratio strongly suggested that the *x*Mn-NFMPP (*x* = 1.5, 1.8, 2.1, 2.4 and 2.7) samples represent a series of isomorphic solid solutions, rather than being a simple physical mixture of Na_4_Fe_3_(PO_4_)_2_P_2_O_7_ and Na_4_Mn_3_(PO_4_)_2_P_2_O_7_. The optimized Na_4_Fe_1.2_Mn_1.8_(PO_4_)_2_P_2_O_7_/C sample was selected for further Ti-doped modification, and the XRD profiles of the *x*Ti-NFMTPP (*x* = 0.1, 0.15, 0.2 and 0.3) samples are shown in Figure 1b. Obviously, the Ti-doped samples exhibited identical crystal structures, crystallinity and phase purity relative to the above Mn-doped compounds. However, the diffraction peaks systematically transitioned to elevated diffraction angles as the Ti substitution content increased, indicating a reduction in the interplanar spacing. The Rietveld refinement results of the *x*Ti-NFMTPP samples are displayed in Figures S6–S9, and the resulting calculated parameters are tabulated within Table S2. With the increasing Ti substitution content, the unit-cell volume of *x*Ti-NFMTPP exhibited a progressive contraction from 1276.41(2) Å^3^ in the pristine 1.8Mn-NFMPP (*x* = 0) to 1272.32(8) Å^3^ (0.1Ti-NFMTPP), ultimately decreasing to 1268.33(4) Å^3^ (0.3Ti-NFMTPP). The systematic unit-cell volume (V) shrinkage can be primarily ascribed to the replacement of Mn^2^^+^ ions (0.80 Å) with smaller-radius Ti⁴^+^ ions (0.68 Å) within the host lattice structure, which induces lattice contraction through ionic size effects [50].

**Figure 2:**
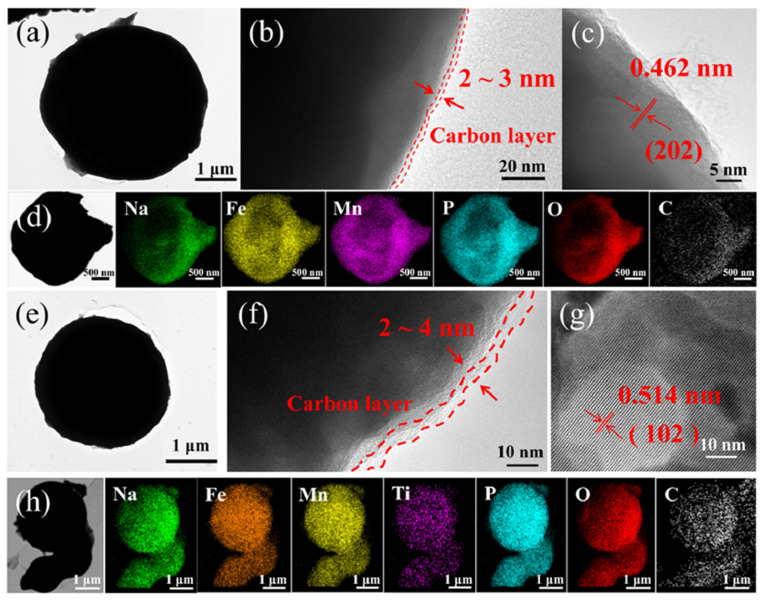
(**a**) TEM image, (**b**,**c**) high-resolution TEM images and (**d**) EDS mapping of 1.8Mn-NFMPP sample; (**e**) TEM image, (**f**,**g**) high-resolution TEM images and (**h**) EDS mapping of 0.1Ti-NFMTPP sample.

The morphology and microstructure of the *x*Mn-NFMPP (*x* = 1.5, 1.8, 2.1, 2.4 and 2.7) and *x*Ti-NFMTPP (*x* = 0.1, 0.15, 0.2 and 0.3) samples were investigated by SEM techniques in Figure S10. Representatives of all the samples were composed of micron-sized spheroidal and/or quasi spheroidal particles sized between 1 and 10 µm and exhibited consistent morphological features for all samples, implying the Mn substitution did not affect the morphology and particle size. However, there were numerous residual pores (or pits) on the surface of these particles, which may be due to the large amount of H_2_O, NO/NO_2_ and CO/CO_2_ generated from the thermal decomposition of the raw materials through high-temperature treatments. In addition, the 1.8Mn-NFMPP and 0.1Ti-NFMTPP samples were carefully selected as the optimal candidates for further detailed composition and structure examination. The analysis of both samples was conducted via TEM coupled with EDS elemental mapping and the results are displayed in Figure 2. Figure 2a–c revealed that the 1.8Mn-NFMPP microspheres (~5 μm diameter) were coated a uniform carbon layer (2~3 nm thick) on the sample surface with an interplanar spacing of 0.462 nm, indexing to the (202) crystallographic plane of NFPP. EDS elemental mapping results confirmed the homogeneous dispersion of Na, Fe, Mn, P, O and C elements in Figure 2d, indicating the effective integration of Mn elements rather than producing other extraneous phases. Similarly, Figure 2e–h illustrated that the 0.1Ti-NFMTPP sample exhibited a spherical particle with a diameter of ~3 μm, featuring a carbon layer of 2~4 nm thickness on the surface. The interplanar spacing of 0.514 nm corresponded to the (102) lattice plane of the NFPP crystal structure. Importantly, EDS mapping clearly revealed the homogeneous dispersion of all constituent elements (including Ti) within the 0.1Ti-NFMTPP particle, which was regarded as the evidence for the substitution of Mn and Ti for Fe elements. Notably, the carbon-coating layer fabricated via the high-temperature pyrolysis of citric acid in the raw materials expectedly mitigated the challenges of intrinsically deficient electronic conductivity and delayed Na^+^ ion diffusion kinetics for NFPP materials. The TG examination was employed to determine the carbon amount of the two samples (Figure S11). The weight fractions of the carbon existing in the 1.8Mn-NFMPP and 0.1Ti-NFMTPP samples were approximately 3.95 wt.% and 3.98 wt.%, respectively. The Raman spectra (Figure S12) exhibited dual broad peaks located at 1570 and 1350 cm^−1^, associated with the crystalline graphitized carbon and amorphous carbon structures [22,34]. The proportion of I_D_/I_G_ was quantified as 1.09 and 1.04 for the 1.8Mn-NFMPP and 0.1Ti-NFMTPP samples, respectively. The two samples had nearly identical content and properties of the carbon-coating layer, attributable to the identical stoichiometric ratio of citric acid in the raw materials and the consistent synthesis process.

**Figure 3:**
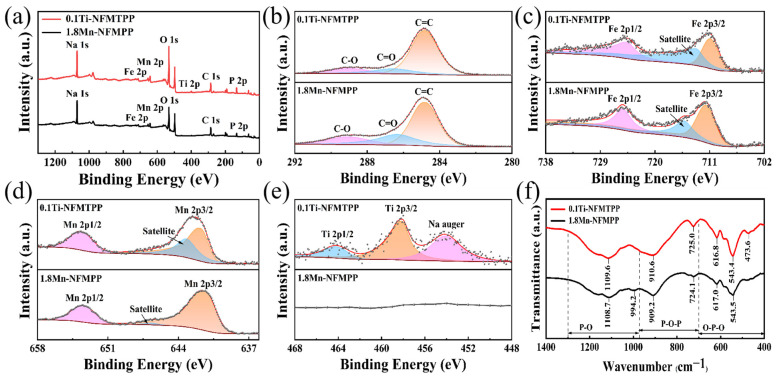
(**a**) XPS wide-scan spectra and high-resolution spectra of (**b**) C 1s; (**c**) Fe 2p; (**d**) Mn 2p and (**e**) Ti 2p; and (**f**) FT-IR spectrum of the 1.8Mn-NFMPP and 0.1Ti-NFMTPP samples.

The XPS technique was applied to probe the oxidation states of the elements in the 1.8Mn-NFMPP and 0.1Ti-NFMTPP samples. The XPS wide-scan spectra of the two samples is depicted in Figure 3a, demonstrating the existence of Na, Fe, Mn, P, O and C components, consistent with the EDS observations. The XPS spectrum in Figure 3b exhibited three prominent peaks located at 288.9, 286.7 and 284.8 eV, corresponding to C–O, C=O and C=C bonds, respectively, with the high proportion of C=C bonds indicating superior electronic conductivity [51]. The high-resolution spectra of Fe 2p exhibited Fe 2p3/2 and Fe 2p1/2 peaks of approximately 711.6 and 725.3 eV in Figure 3c, respectively, which confirmed the existence of Fe^2+^. The fitted data of Mn 2p displayed obvious dual bands of the Mn 2p3/2 and Mn 2p1/2 spintronic orbitals at 641.6 and 653.4 eV in Figure 3d, respectively, suggesting that Mn existed mainly in the Mn^2+^ state. Broad satellite peaks were additionally detected at a higher binding energy to each principal peak, resulting from electron redistribution during the photo-emission process, which commonly occurs in XPS spectra [52]. As shown in Figure 3e, the high-definition spectra of Ti 2p exhibited characteristic binding energies located at 458.2 and 464.1 eV, correspondingly assigned to Ti 2p3/2 and Ti 2p1/2 peaks. The spectra only corresponded to a very weak peak in the XPS wide-scan spectra of the 0.1Ti-NFMTPP sample, indicating trace-level Ti incorporation within the host crystal lattice. Additionally, the FT-IR analysis of the 1.8Mn-NFMPP and 0.1Ti-NFMTPP samples (Figure 3f) demonstrated distinctive absorption bands spanning the spectral regions 975~1300 and 400~700 cm^−1^, which are associated with P-O elongation vibrations and the O-P-O angular distortions of PO_4_^3−^ tetrahedra, respectively. The peaks positioned at 725 and 910 cm^−1^ belonged to balanced and unbalanced P-O-P vibrations in P_2_O_7_^4−^ structural units, which also coincided well with results from previous reports in the literature [53].

**Figure 4:**
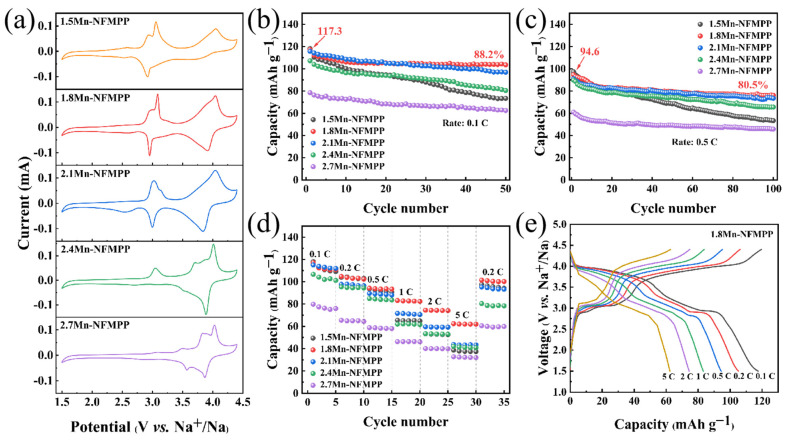
(**a**) CV curves; cycle durability at (**b**) 0.1 C and (**c**) 0.5 C; and (**d**) rate performance of *x*Mn-NFMPP (*x* = 1.5, 1.8, 2.1, 2.4 and 2.7) samples. (**e**) Charge–discharge curves of 1.8Mn-NFMPP sample at different rates.

The electrochemical performance of all the *x*Mn-NFMPP (*x* = 1.5, 1.8, 2.1, 2.4 and 2.7) samples as cathodes of SIBs was evaluated by CV within 1.5 ~ 4.4 V (vs. Na^+^/Na) at 0.1 mV s^−1^ and galvanostatic charge–discharge tests to research the effects of various Mn content as doping modifications. All samples exhibited similar multiple oxidation/reduction peaks as shown in Figure 4a, which was related to the stepwise intercalation/deintercalation process of Na^+^ in these samples. The dual distinct pairs of oxidation/reduction peaks positioned at 3.08/2.96 and 4.03/3.92 V (vs. Na^+^/Na) aligned with the sequential Fe^3+^/Fe^2+^ and Mn^3+^/Mn^2+^ redox peaks. The redox potentials were consistent with a previous report [54], indicating a reversible Na^+^ intercalation/deintercalation reaction in the NFPP lattice. The slight differences in the redox potential and square of the peak current (*i*_P_^2^) among these samples may be assigned to the variations in the Mn substitution content, which potentially influenced the Na^+^ diffusion coefficient and electronic conductivity [55]. However, the redox couples at 3.08/2.96 V (vs. Na^+^/Na) gradually weakened and eventually disappeared as the Mn substitution content increased. Instead, the emergence of additional redox peaks, particularly detected at 3.84/3.58 V (vs. Na^+^/Na), became more prominent in the 2.7Mn-NFMPP sample, which corresponded to the Mn^3^^+^/Mn^2^^+^ redox reaction. The peaks were related to the intercalation/deintercalation of Na^+^ in the NFPP lattice, indicating the chemical environment of Na^+^ is significantly altered by the Mn substitution, leading to its insertion/extraction at a higher voltage [56].

The cyclic performance of all the *x*Mn-NFMPP (*x* = 1.5, 1.8, 2.1, 2.4 and 2.7) samples were investigated at 0.1 C and 0.5 C (1 C = 129 mAh g^−1^) and are displayed in Figure 4b,c. From the data, the 1.8Mn-NFMPP sample exhibited the highest reversible capacities, delivering an initial capacity of 117.3 mAh g^−1^ at 0.1 C with 88.2% retention after 50 cycles; at 0.5 C, the sample achieved 94.6 mAh g^−1^ with 80.5% retention over 100 cycles. The reversible capacities of the 1.5Mn-NFMPP, 1.8Mn-NFMPP, 2.1Mn-NFMPP, 2.4Mn-NFMPP and 2.7Mn-NFMPP samples arrived at 73.5, 103.5, 97.0, 80.5 and 62.5 mAh g^−1^ over 50 cycles at 0.1 C, and 53.4, 76.2, 73.5, 65.6 and 45.8 mAh g^−1^ after 100 cycles at 0.5 C, respectively. The rate performance of all the materials at different C-rates from 0.1 to 5 C were compared in Figure 4d. As demonstrated, the 1.8Mn-NFMPP sample also achieved the highest rate capability, with capacities of 117.0, 104.7, 94.1, 83.1, 74.5 and 62.4 mAh g^−1^ at 0.1, 0.2, 0.5, 1, 2 and 5 C, respectively. Furthermore, the reversible capacity of the sample could be recovered to 101.4 mAh g^−1^ when the current density reverted to 0.2 C. Even at a 5 C rate, the 1.8Mn-NFMPP sample could deliver a capacity of 62.4 mAh g^−1^, significantly surpassing the values from other samples: 38.7 mAh g^−1^ for 1.5Mn-NFMPP, 43.7 mAh g^−1^ for 2.1Mn-NFMPP, 42.1 mAh g^−1^ for 2.4Mn-NFMPP and 32.8 mAh g^−1^ for 2.7Mn-NFMPP). The charge–discharge voltage curves of the 1.8Mn-NFMPP sample at varying C-rates are presented from the second cycle of rate performance in Figure 4e. The electrode demonstrated a distinct electrochemical voltage platform positioned at 3.0/2.9 and 4.0/3.9 V (vs. Na^+^/Na) in agreement with its CV curve. At the 0.1 C rate, the mean discharge voltage was ~3.6 V (vs. Na^+^/Na). With an escalating current density, the specific capacity gradually decreased and the voltage platform shortened. It is notable that the Na_4_Fe_1.2_Mn_1.8_(PO_4_)_2_P_2_O_7_ material, with a Mn/Fe molar ratio of 6:4, exhibited superior electrochemical properties among the *x*Mn-NFMPP (*x* = 1.5, 1.8, 2.1, 2.4 and 2.7) samples. The observation is not only consistent with previously reported results but also aligns with the Mn/Fe stoichiometry (6:4) commonly employed in commercial LiFe_1-*x*_Mn*_x_*PO_4_ cathodes for LIBs, thereby highlighting the significance of the specific Mn/Fe ratio in achieving optimal electrochemical performance [57].

**Figure 5:**
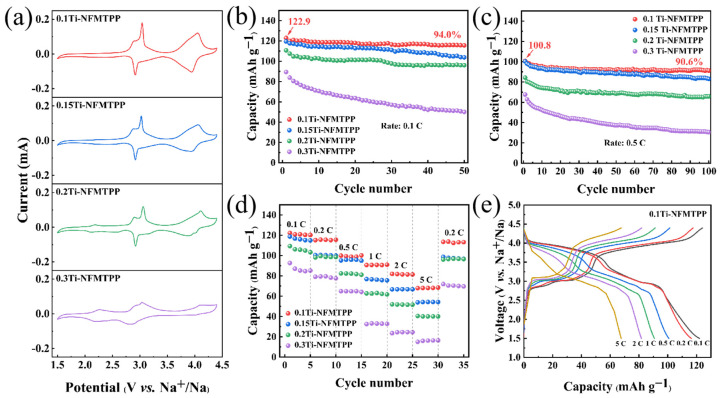
(**a**) CV curves; cycle durability at (**b**) 0.1 C and (**c**) 0.5 C; and (**d**) rate performance of *x*Ti-NFMTPP (*x* = 0.1, 0.15, 0.2 and 0.3) samples. (**e**) Charge–discharge curves of 0.1Ti-NFMTPP sample at different rates.

Based on the aforementioned discussion, the Na_4_Fe_1.2_Mn_1.8_(PO_4_)_2_P_2_O_7_ material exhibited superior electrochemical properties in the various *x*Mn-NFMPP samples. However, its poor cycling stability remained unsatisfactory, necessitating further optimization to improve its long-term cycling performance. To address this issue, a series of Ti-doped *x*Ti-NFMTPP (*x* = 0.1, 0.15, 0.2 and 0.3) samples were synthesized and investigated to boost the electrochemical performance of 1.8Mn-NFMPP. The electrochemical properties of all the *x*Ti-NFMTPP materials were further characterized via CV and galvanostatic charge/discharge tests. Figure 5a illustrates the CV profiles acquired at 0.1 mV s^−1^. Similar to the 1.8Mn-NFMPP sample, the Ti-doped *x*Ti-NFMTPP samples demonstrated dual prominent redox couples at 3.03/2.93 and 4.05/3.94 V (vs. Na^+^/Na), relating to the sequential Fe^3+^/Fe^2+^ and Mn^3+^/Mn^2+^ redox reactions. Nevertheless, the redox peak at 2.26/2.13 V (vs. Na^+^/Na) became increasingly pronounced with the increasing Mn substitution content, particularly in the 0.3Ti-NFMTPP samples, indicating the Ti^4+^/Ti^3+^ redox reaction [58].

To further characterize the influence of Ti doping on the electrochemical performance of the 1.8Mn-NFMPP sample, the cyclic durability of the *x*Ti-NFMTPP (*x* = 0.1, 0.15, 0.2 and 0.3) samples at 0.1 C and 0.5 C were investigated and are presented in Figure 5b,c. Obviously, the 0.1Ti-NFMTPP sample demonstrated excellent reversible capacity and cyclic durability relative to the additional three samples when the substitution amount for Ti doping was 0.1. The sample delivered a capacity of 122.9 mAh g^−1^ under 0.1 C, retaining 94.0% of the initial capacity through 50 cycles, while delivering 100.8 mAh g^−1^ at 0.5 C with 90.6% capacity retention after 100 cycles. Compared with 1.8Mn-NFMPP sample, the cyclic durability of 0.1Ti-NFMTPP was significantly improved, which may be attributed to the fact that Ti doping effectively alleviates the Jahn–Teller distortion and suspends Mn dissolution. Additionally, the specific discharge capacities of the 0.15Ti-NFMTPP, 0.2Ti-NFMTPP and 0.3Ti-NFMTPP samples achieved 103.8, 96.1 and 50.1 mAh g^−1^ at 0.1 C over 50 cycles, and 83.0, 65.9 and 30.6 mAh g^−1^ at 0.5 C over 100 cycles, respectively. The 0.1Ti-NFMTPP sample showed better rate performance as shown in Figure 5d, which delivered 122.2, 115.2, 99.9, 90.7, 82.0 and 67.9 mAh g^−1^ at 0.1, 0.2, 0.5, 1, 2 and 5 C, respectively. When returned to 0.2 C, the sample demonstrated a reversible capacity of 113.5 mAh g^−1^. Also, the sample demonstrated a superior specific discharge capacity relative to the additional three samples under a high 5 C rate, with values of 54.0 mAh g^−1^ for 0.15Ti-NFMTPP, 40.5 mAh g^−1^ for 0.2Ti-NFMTPP and 15.1 mAh g^−1^ for 0.3Ti-NFMTPP. The voltage profiles of the 0.1Ti-NFMTPP sample at different C-rates are displayed in Figure 5e. The voltage profiles of 0.1Ti-NFMTPP exhibited two distinct charge–discharge plateaus, which aligned closely with those observed for 1.8Mn-NFMPP. Notably, the 0.1Ti-NFMTPP sample demonstrated significantly higher specific capacities than its predecessor 1.8Mn-NFMPP. For example, the 0.1Ti-NFMTPP electrode exhibited a superior capacity of 116.3 mAh g^−1^ at 0.2 C and 100.8 mAh g^−1^ at 0.5 C, whereas the 1.8Mn-NFMPP sample achieved a specific capacity of 105.1 mAh g^−1^ at 0.2 C and 94.6 mAh g^−1^ at 0.5 C. These results illustrate that strategic Ti doping constitutes a viable approach to boost the electrochemical properties of Mn-doped NFPP, achieving mitigation of the Jahn-Teller distortion and Mn dissolution, thereby significantly improving the structural stability and cycle durability.

**Figure 6:**
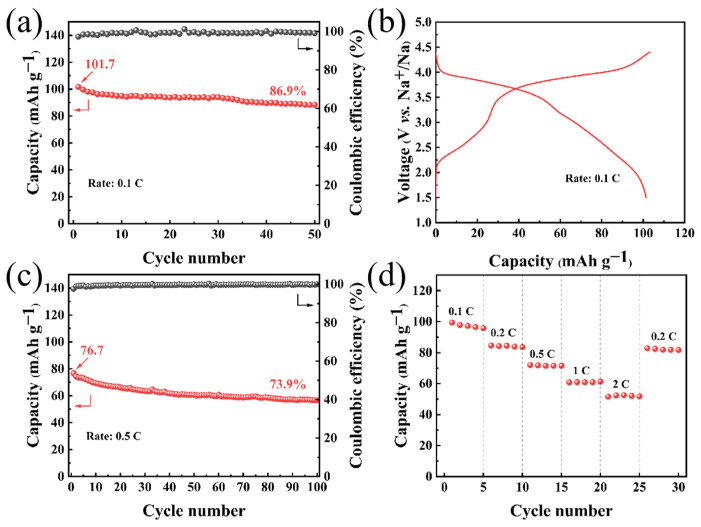
(**a**) Cycle durability at 0.1 C; (**b**) First cycle charge–discharge curves; (**c**) Cycle durability at 0.5 C and (**d**) Rate performance of the coin-type full cell.

To further assess the prospects of Ti-doped 0.1Ti-NFMTPP materials for practical applications, coin-type full cells were assembled employing commercial hard carbon as the anode and the 0.1Ti-NFMTPP sample as the cathode. The characterization and electrochemical properties of the hard carbon are displayed in Figure S13. The hard carbon electrodes demonstrated satisfactory electrochemical properties in the half-cell configuration, delivering a capacity of 297.5 mAh g^−1^ at 0.1 C (1 C = 300 mAh g^−1^) within 0.01~3 V, retaining 96% capacity over 100 cycles. Based on these results, the mass ratio of the hard carbon/Ti-doped 0.1Ti-NFMTPP was optimized to 1:2.4 for the capacity balancing of the anode and cathode. As illustrated in Figure 6a,b, the full cell operated within the potential window of 1.5~4.4 V, delivering an initial specific capacity of 101.7 mAh g^−1^ (relative to 0.1Ti-NFMTPP mass), maintaining 86.9% of its initial capacity at 0.1 C (1 C = 129 mAh g^−1^) over 50 cycles and exhibiting a mean operating voltage around 3.5 V. When examined at an elevated 0.5 C as shown in Figure 6c, the full cell merely attained a capacity of 76.7 mAh g^−1^ with 73.9% capacity retention over 100 cycles. However, the rate capability shown in Figure 6d revealed suboptimal performance with reversible capacities of 84.6, 60.9 and 51.6 mAh g^−1^ at 0.2, 1 and 2 C, respectively, indicating significant challenges in high-rate performance. The intrinsic causation of this phenomenon in the full-cell system remains unclear, possibly due to the poor high-rate performance caused by the large particle size (~5 μm) of the sample. Potential contributing factors include the limited electrochemical capabilities of the hard carbon anode, polarization of electrode materials in the non-aqueous electrolyte, or detachment of active material from current collectors. We propose that the electrochemical performance of full SIBs systems employing 0.1Ti-NFMTPP could be further optimized through the implementation of improved anode materials and tailored electrolyte compositions.

## 4. Conclusions

In conclusion, we effectively synthesized a series of Mn-doped Na_4_Fe_3-*x*_Mn*_x_*(PO_4_)_2_P_2_O_7_ and Ti-doping Na_4_Fe_1.2_Mn_1.8-*x*_Ti*_x_*(PO_4_)_2_P_2_O_7_ materials via a facile spray-drying method. XRD analysis confirmed that Mn and/or Ti doping maintained the intrinsic crystal lattice of NFPP without inducing impurities. The optimized 1.8Mn-NFMPP sample initially delivered an initial capacity of 117.3 mAh g^−1^, maintaining 88.2% of its capacity over 50 cycles at 0.1 C with an enhanced operating voltage of ~3.6 V. However, it exhibited unsatisfactory cycling stability. To address this issue, Ti doping was strategically introduced into the 1.8Mn-NFMPP sample to enhance its electrochemical properties. Subsequent electrochemical testing revealed that the optimized Mn/Ti co-doped 0.1Ti-NFMTPP material exhibited superior electrochemical performance compared to the 1.8Mn-NFMPP, delivering a specific capacity of 122.9 mAh g^−^¹ at 0.1 C, retaining 94.0% of its initial capacity over 50 cycles. Furthermore, a coin-type full cell assembled with the optimized cathode and hard carbon as the anode demonstrated superior electrochemical performance, substantiating its viability for practical applications. These results highlight that optimal Ti doping effectively mitigates the Jahn–Teller distortion and Mn dissolution, offering a dual-functional strategy to improve the structural stability and cycling performance of Mn-doped NFPP systems.

## Data Availability

The original contributions presented in the study are included in the article/Supplementary Materials, further inquiries can be directed to the corresponding authors.

## Supplementary Materials

The following supporting information can be downloaded at: https://www.mdpi.com/article/10.3390/nano15080581/s1. Figures S1–S9: The Rietveld refinement of the observed XRD patterns for *x*Mn-NFMPP (*x* = 1.5, 1.8, 2.1, 2.4 and 2.7) and *x*Ti-NFMTPP (*x* = 0.1, 0.15, 0.2 and 0.3) samples, respectively. Figure S10: SEM images of *x*Mn-NFMPP (*x* = 1.5, 1.8, 2.1, 2.4 and 2.7) and *x*Ti-NFMTPP (*x* = 0.1, 0.15, 0.2 and 0.3) samples, respectively. Figure S11: TG curves of the 1.8Mn-NFMPP and 0.1Ti-NFMTPP samples. Figure S12: Raman spectrums of the 1.8Mn-NFMPP and 0.1Ti-NFMTPP samples. Figure S13: XRD pattern, SEM image, the first four cycled charge-discharge curves and the cycling performance of hard carbon half-cell at 0.1C. Tables S1–S2: The calculated lattice parameters obtaining from the Rietveld refinement of the *x*Mn-NFMPP (*x* = 1.5, 1.8, 2.1, 2.4 and 2.7) and *x*Ti-NFMTPP (*x* = 0.1, 0.15, 0.2 and 0.3) samples, respectively.
