# Supplementary material for: Ti-Doped, Mn-Based Polyanionic Compounds of Na4Fe1.2Mn1.8(PO4)2P2O7 for Sodium-Ion Battery Cathode
Source: Nanomaterials (Basel). 2025 Apr 11;15(8):581. doi: 10.3390/nano15080581 (PMC12029225; doi:10.3390/nano15080581)
Supplement: Supplementary file 1 [file nanomaterials-15-00581-s001.zip › nanomaterials-3571535-supplementary.pdf]

Supporting Information

Ti-Doped Polyanionic Mn-Based Compounds of $Na_4Fe_{1.2}Mn_{1.8}(PO_4)_2P_2O_7$ for Sodium-Ion Battery Cathode

Hualin Li,[a] Gang Pang[a] *, Weilong Zhang,[a] Qingan Zhang[a] *, Linrui Hou[b] and Changzhou Yuan[b] *

[a] School of Materials Science and Engineering and Key Laboratory of Efficient Conversion and Solid-state Storage of Hydrogen & Electricity of Anhui Province, Anhui University of Technology, Ma'anshan 243002, China.
[b] School of Materials Science & Engineering, University of Jinan, Jinan 250022, P. R. China

* Correspondence: panggang22@ahut.edu.cn; qazhang@ahut.edu.cn; mse_yuancz@ujn.edu.cn;

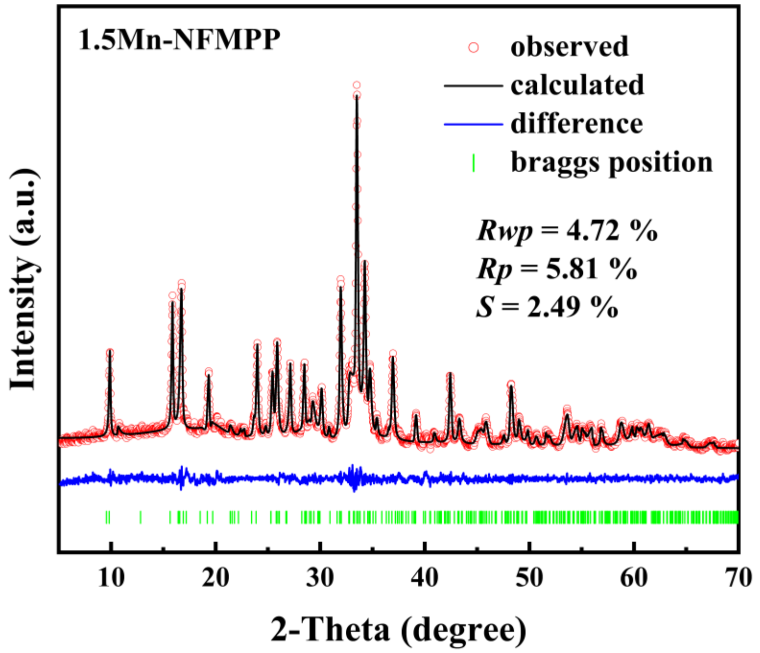

Figure S1 The Rietveld refinement of the observed XRD patterns for 1.5Mn-NFMPP.

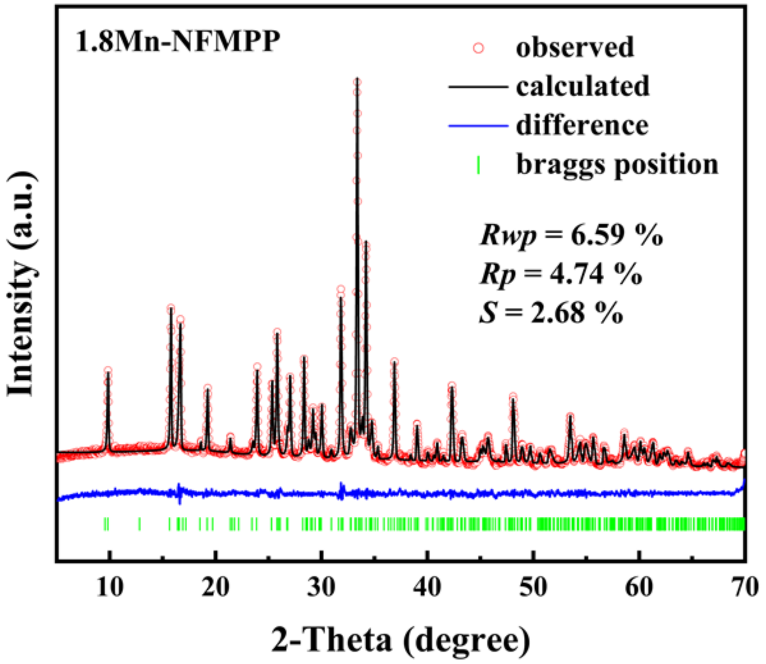

Figure S2 The Rietveld refinement of the observed XRD patterns for 1.8Mn-NFMPP.

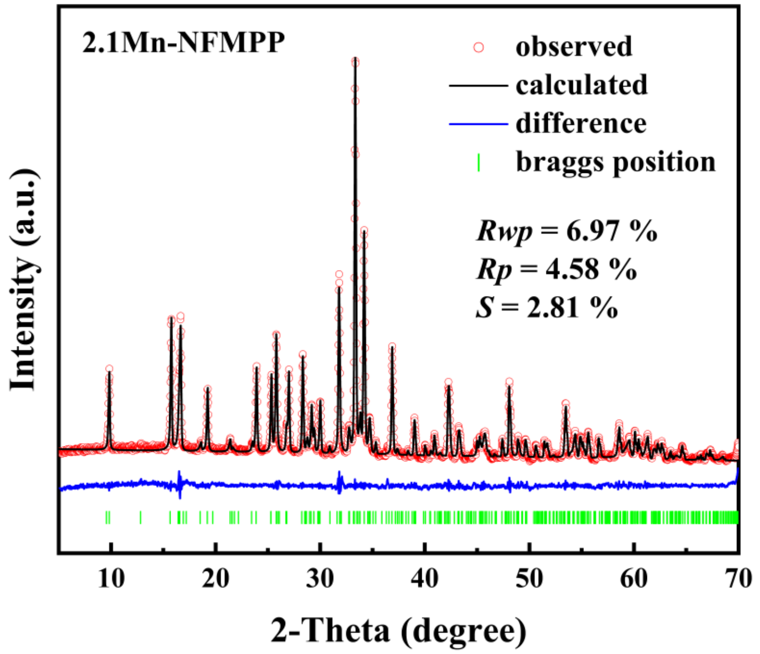

Figure S3 The Rietveld refinement of the observed XRD patterns for 2.1Mn-NFMPP.

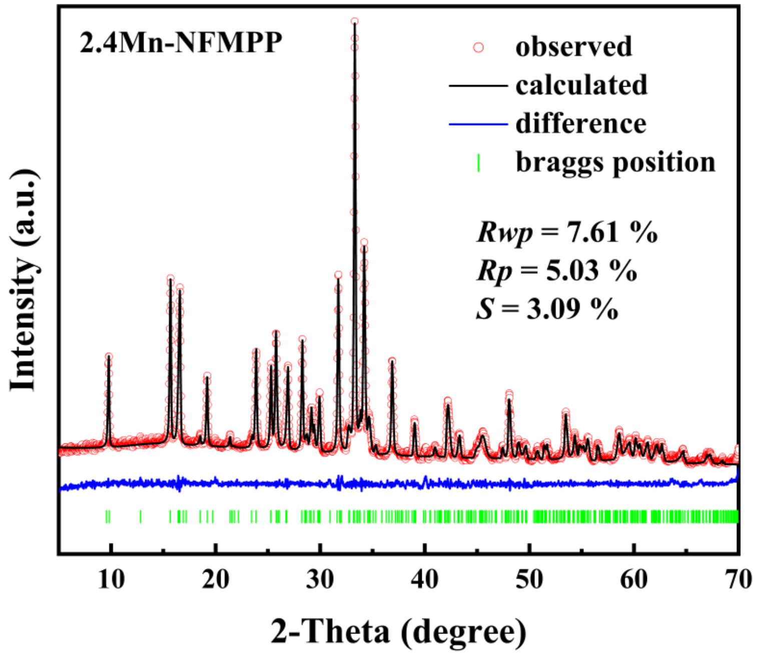

Figure S4 The Rietveld refinement of the observed XRD patterns for 2.4Mn-NFMPP.

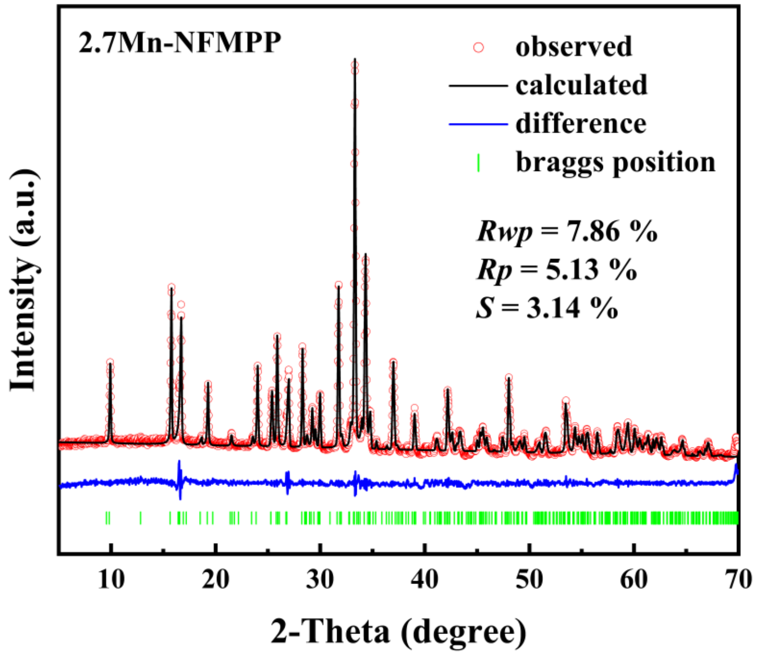

Figure S5 The Rietveld refinement of the observed XRD patterns for 2.7Mn-NFMPP.

Table S1 The calculated lattice parameters obtaining from the Rietveld refinement of the *x*Mn-NFMPP (*x* = 1.5, 1.8, 2.1, 2.4 and 2.7) samples.

sample	lattice parameters			unit-cell volume
	a (Å)	b (Å)	c (Å)	($Å^3$)
1.5Mn-NFMPP	18.0239(2)	6.5931(3)	10.7127(4)	1273.03(8)
1.8Mn-NFMPP	18.0307(2)	6.6012(7)	10.7238(4)	1276.41(2)
2.1Mn-NFMPP	18.0376(1)	6.6125(9)	10.7351(7)	1280.44(1)
2.4Mn-NFMPP	18.0429(5)	6.6210(7)	10.7589(2)	1285.29(9)
2.7Mn-NFMPP	18.0511(6)	6.6289(6)	10.7721(4)	1288.99(8)

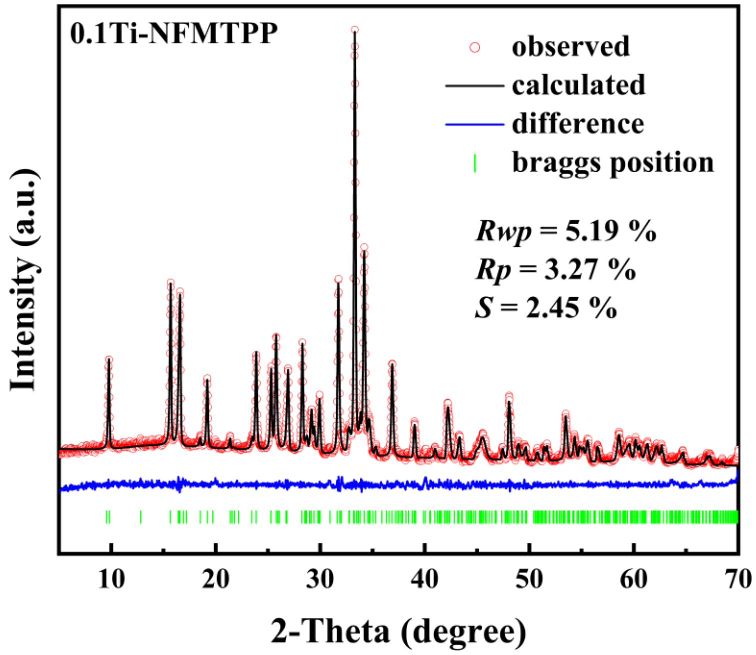

Figure S6 The Rietveld refinement of the observed XRD patterns for 0.1Ti-NFMTPP.

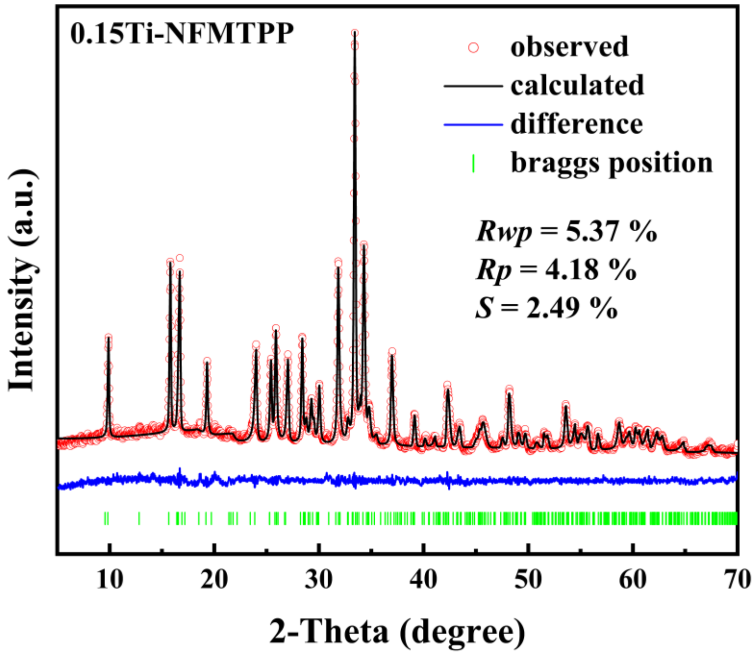

Figure S7 The Rietveld refinement of the observed XRD patterns for 0.15Ti-NFMTPP.

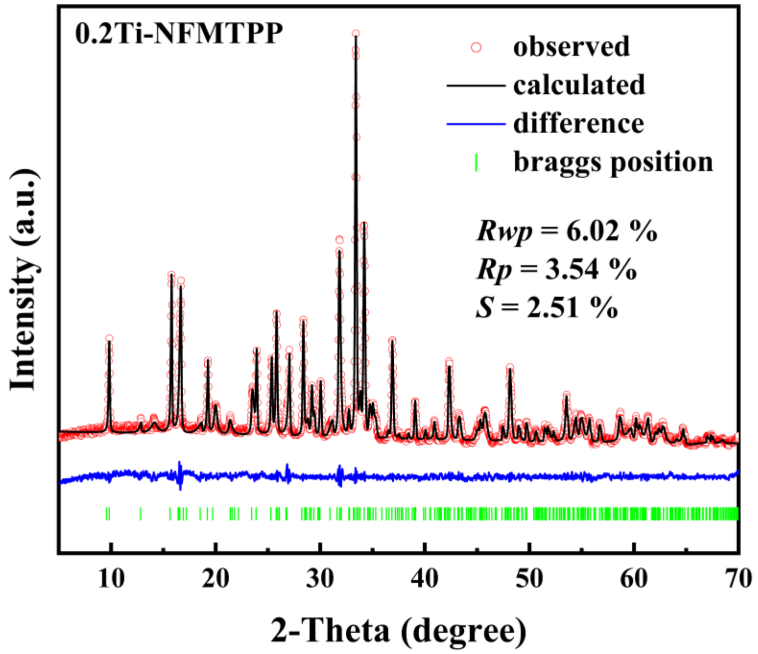

Figure S8 The Rietveld refinement of the observed XRD patterns for 0.2Ti-NFMTPP.

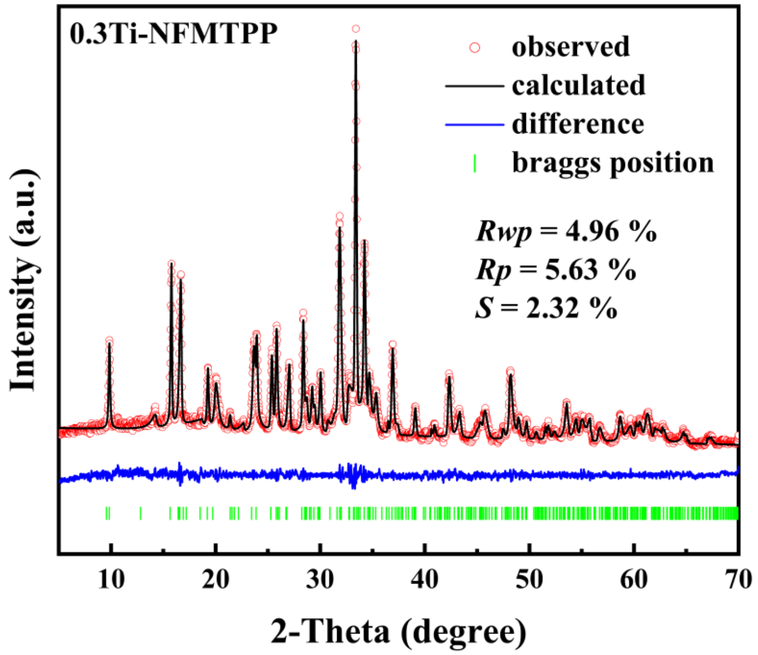

Figure S9 The Rietveld refinement of the observed XRD patterns for 0.3Ti-NFMTPP.

Table S2 The calculated lattice parameters obtaining from the Rietveld refinement of the *x*Ti-NFMTPP (*x* = 0.1, 0.15, 0.2 and 0.3) samples.

sample	lattice parameters			unit-cell volume
	a (Å)	b (Å)	c (Å)	(Å^3)
0.1Ti-NFMTPP	17.9860(8)	6.5956(2)	10.7252(4)	1272.32(8)
0.15Ti-NFMTPP	17.9772(7)	6.5881(3)	10.7285(7)	1270.65(5)
0.2Ti-NFMTPP	17.9711(8)	6.5817(2)	10.7305(4)	1269.22(2)
0.3Ti-NFMTPP	17.9640(9)	6.5805(9)	10.7291(1)	1268.33(4)

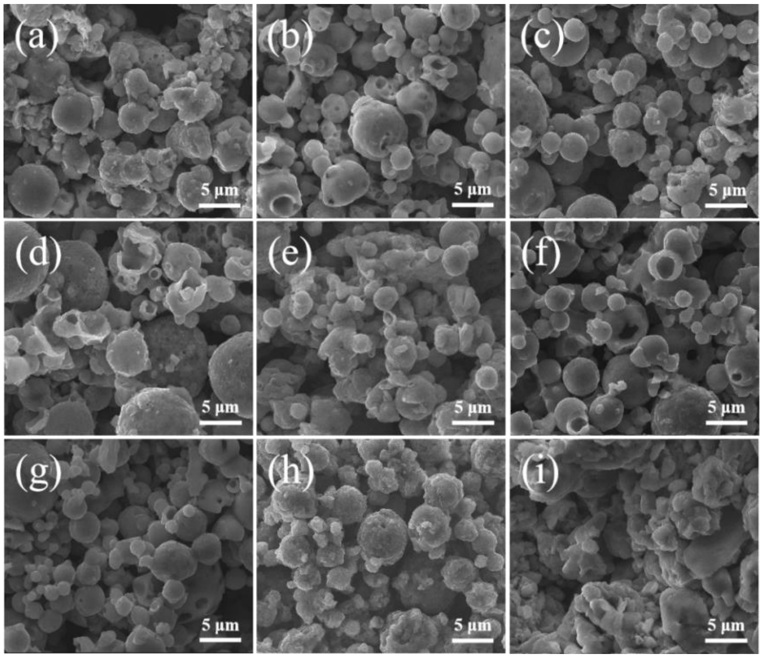

Figure S10 SEM images of **(a~e)** *x*Mn-NFMPP (*x* = 1.5, 1.8, 2.1, 2.4 and 2.7) and **(f~i)** *x*Ti-NFMTPP (*x* = 0.1, 0.15, 0.2 and 0.3) samples, respectively.

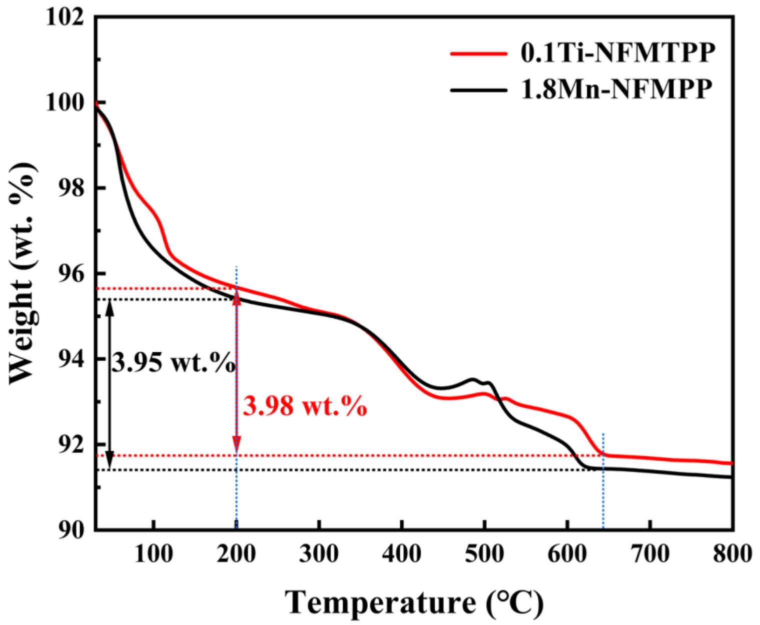

Figure S11 TG curves of the 1.8Mn-NFMPP and 0.1Ti-NFMTPP samples.

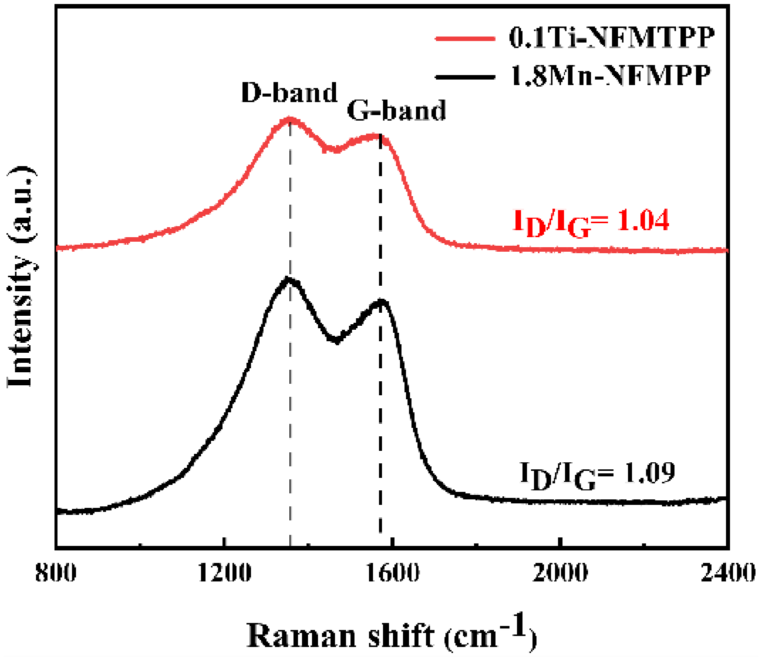

Figure S12 Raman spectrums of the 1.8Mn-NFMPP and 0.1Ti-NFMTPP samples.

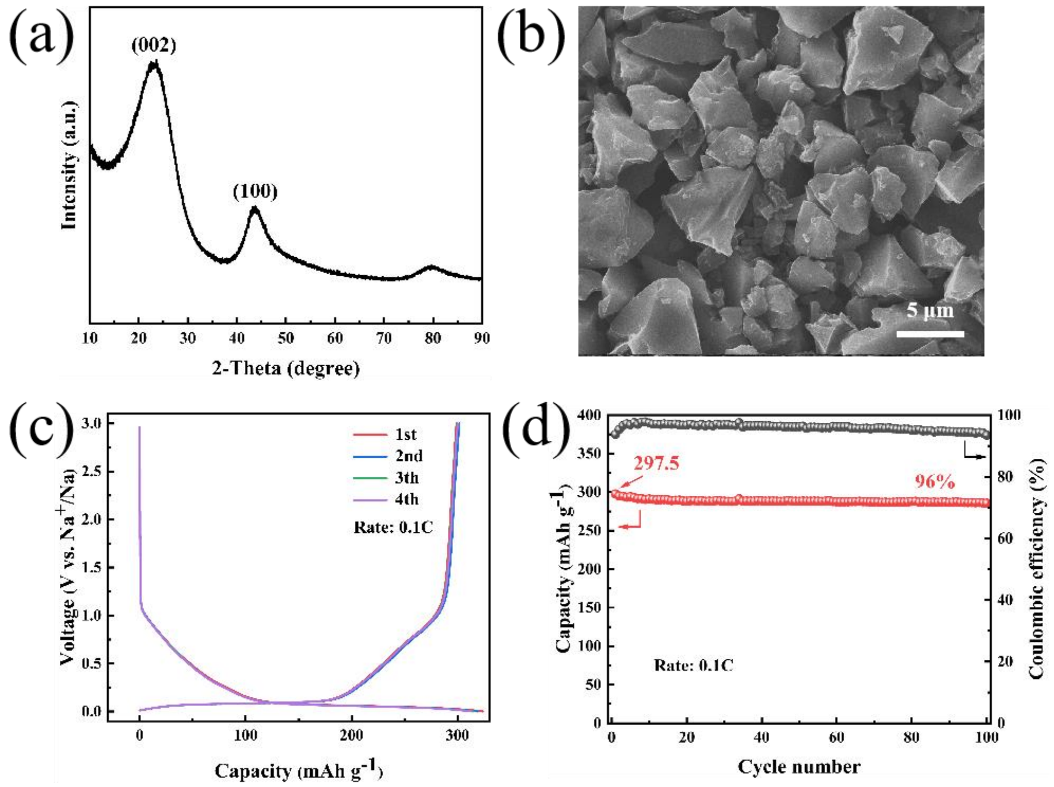

Figure S13 (a) XRD pattern, **(b)** SEM image, **(c)** the first four cycled charge-discharge curves and **(d)** the cycling performance of hard carbon half-cell at 0.1C.
